# Multiomics and bioinformatics identify differentially expressed effectors in the brain of *Toxoplasma gondii* infected masked palm civet

**DOI:** 10.3389/fcimb.2023.1267629

**Published:** 2023-09-25

**Authors:** Hao Yuan, Tiantian Jiang, Wei-Dong Zhang, Zipeng Yang, Shengjun Luo, Xiaohu Wang, Xiaojing Zhu, Shuting Qi, Yasser S. Mahmmod, Xiu-Xiang Zhang, Zi-Guo Yuan

**Affiliations:** ^1^ Guangdong Provincial Key Laboratory of Zoonosis Prevention and Control, College of Veterinary Medicine, South China Agricultural University, Guangzhou, China; ^2^ Key Laboratory of Zoonosis of Ministry of Agriculture and Rural Affairs, South China Agricultural University, Guangzhou, Guangdong, China; ^3^ Department of Pediatrics, School of Medicine, University of California, La Jolla, San Diego, CA, United States; ^4^ South China Agricultural University Hospital, Guangzhou, China; ^5^ Institute of Animal Health, Guangdong Academy of Agricultural Sciences, Guangzhou, China; ^6^ Infectious Diseases, Department of Animal Medicine, Faculty of Veterinary Medicine, Zagazig University, Zagazig, Egypt; ^7^ Veterinary Sciences Division, Faculty of Health Sciences, Higher Colleges of Technology, Abu Dhabi, United Arab Emirates; ^8^ College of Agriculture, South China Agricultural University, Guangzhou, China

**Keywords:** Toxoplasma gondii, masked palm civet, proteomics, transcriptome, glioblastoma

## Abstract

**Introduction:**

The masked palm civet (*Paguma larvata*) serves as a reservoir in transmitting pathogens, such as *Toxoplasma gondii*, to humans. However, the pathogenesis of *T. gondii* infection in masked palm civets has not been explored. We studied the molecular changes in the brain tissue of masked palm civets chronically infected with *T. gondii* ME49.

**Methods:**

The differentially expressed proteins in the brain tissue were investigated using iTRAQ and bioinformatics.

**Results:**

A total of 268 differential proteins were identified, of which 111 were upregulated and 157 were downregulated. KEGG analysis identified pathways including PI3K-Akt signaling pathway, proteoglycans in cancer, carbon metabolism, T-cell receptor signaling pathway. Combing transcriptomic and proteomics data, we identified 24 genes that were differentially expressed on both mRNA and protein levels. The top four upregulated proteins were REEP3, REEP4, TEP1, and EEPD1, which was confirmed by western blot and immunohistochemistry. KEGG analysis of these 24 genes identified signaling cascades that were associated with small cell lung cancer, breast cancer, Toll-like receptor signaling pathway, Wnt signaling pathways among others. To understand the mechanism of the observed alteration, we conducted immune infiltration analysis using TIMER databases which identified immune cells that are associated with the upregulation of these proteins. Protein network analysis identified 44 proteins that were in close relation to all four proteins. These proteins were significantly enriched in immunoregulation and cancer pathways including PI3K-Akt signaling pathway, Notch signaling pathway, chemokine signaling pathway, cell cycle, breast cancer, and prostate cancer. Bioinformatics utilizing two cancer databases (TCGA and GEPIA) revealed that the four genes were upregulated in many cancer types including glioblastoma (GBM). In addition, higher expression of REEP3 and EEPD1 was associated with better prognosis, while higher expression of REEP4 and TEP1 was associated with poor prognosis in GBM patients.

**Discussion:**

We identified the differentially expressed genes in the brain of *T. gondii* infected masked palm civets. These genes were associated with various cellular signaling pathways including those that are immune- and cancer-related.

## 1 Introduction

*Toxoplasma gondii* can invade virtually any organs in animals and humans, and its preferred sites are the brain, eyes, heart, lungs and muscles (Kochanowsky and Koshy, 2018). Under the attack of the host immune system, *T. gondii* retreats to neural and muscular tissues and forms tissue cysts, which remain for the duration of host lifespan (Menard et al., 2019). *T. gondii* can invade the host’s central nervous system (CNS) and cause encephalitis (Feustel et al., 2012). *T. gondii* infection of the CNS has been associated with Alzheimer’s disease, paralysis, epilepsy, glioblastoma, brain tumors, and other common neurological disorders (Schlüter and Barragan, 2019; Hodge et al., 2021; Nayeri et al., 2021; Virus et al., 2021). Likely due to infection of CNS, hosts exhibit behavioral changes, such as decreased locomotor activity, impaired learning and memory abilities, and reduced awareness of felids in the case of rodents (Markus et al., 2021). The current known entry routes of *T. gondii* into the brain are crossing the blood-brain barrier and the Trojan Horse mechanism (Feustel et al., 2012). Felids are the sole definitive hosts of *T. gondii*. Felids release oocysts into the environment which contributes to the infection in a variety of intermediate hosts (Desmettre, 2020).

The masked palm civet (*Paguma larvata*, Mammalia: Carnivora: Viverridae) mainly inhabits forests, caves, and tree caves. In the wild, masked palm civets feed on wild fruits and grains, insects, frogs, birds, eggs, and mice (Wang and Eaton, 2007; Liu et al., 2022). Due to its wide range of food sources, masked palm civet can harbor a wide array of infectious diseases. Because of its valuable fur and exotic meat, the masked palm civet is poached in many economically underdeveloped areas (Li et al., 2003; Hou et al., 2016).

After the outbreak of SARS in Asian countries in 2003, researchers isolated SARS-CoV from masked palm civets, confirming that masked palm civets are a direct source of SARS-CoV in humans (Li et al., 2005; Shi and Hu, 2008). In addition, the masked palm civet is a reservoir of numerous harmful viruses, bacteria, and parasites, including, reoviruses, rabies virus, *Toxoplasma gondii*, Yersinia pseudotuberculosis, and Salmonella enterica (Lee et al., 2011; Matsumoto et al., 2011; Li et al., 2015; Hou et al., 2016). In areas where the animal is hunted, masked palm civets serve as an important vehicle for disease transmission to humans. Understanding the disease pathogenesis in masked palm civets is a priority of curtailing disease transmission to humans.

In our previous study, we took the brain tissues of *T. gondii* infected masked palm civets and conducted transcriptomic studies (Yuan et al., 2022). We identified differentially expressed genes (e.g., CCL28, CCL23, TLR1, TLR4, CCL5, CASP8) that were enriched in immune regulatory pathways, including chemokine signaling pathway, TLR signaling pathway, and PI3K-Akt signaling pathway. Here, we conducted proteomics and incorporated previous published transcriptomic data to comprehensively analyze the molecular changes in the brains of infected mask palm civets. The differentially expressed proteins (DEPs) were identified in *T. gondii* infected masked palm civets. Notably, GBM associated DEPs were identified. Immune infiltration and a protein network analysis were conducted to explore immune cells and proteins that are directly associated with these DEPs.

## 2 Materials and methods

### 2.1 Experimental animals and *T. gondii* strain

The six, 3 to 4-month-old masked palm civets were provided by the special breeding base in Shaoguan (Guangdong, China) and kept under controlled conditions in an animal facility in South China Agricultural University. Prior to *T. gondii* infection, animal sera were screened *via* ELISA for feline panleukopenia virus, feline immunodeficiency virus, and feline coronavirus. All serological tests showed negative results.

The animals were separated into the experimental group (n=5) and control group (n=1). *T. gondii* ME49 was acquired from the South China Agricultural University’s Parasitology Lab (Guangdong, China). Kunming mice were intraperitoneally injected with ME49 tachyzoites and 10 days later, brains were harvested for cyst preparation. The cysts were enumerated under the microscope using a hemacytometer and diluted to 10^3^ cysts per microliter in PBS. The experimental group was inoculated with 1mL of 10^3^ cysts *via* intraperitoneal injection, and the control group was inoculated with 1 mL of sterile PBS *via* the same infection route. A modified agglutination test (MAT) was performed to confirm *T. gondii* infection in the masked palm civets. After 90 days, brain tissues were collected aseptically from the experimental and control groups and stored at -80°C.

### 2.2 Amplification of *T. gondii* genes and protein extraction

DNA was extracted from the collected brain tissues of masked palm civets, *T. gondii* infection was detected targeting B1 gene (194bp) and repeated sequence Rep-529 (529bp) using primers (F: 5’-TCTTTAAAGCGTTCGTGTC-3’, R: 5’-GGAACTGCATCCGTTCATGAG-3’) and (F: 5’-CGCTGCAGGGAGGAAGACGAAAGTTG-3’, R: 5’-CGCTGCAGACACAGTGCATCTGGATT-3’), respectively.

For protein extraction, 100mg of brain tissue was taken and grinded in liquid nitrogen. A RIPA lysis buffer was added (50mM Tris, 150 mM NaCl, 1% TritonX-100, 1%SDS) and incubated at 4°C for 30mins. The mixture was centrifuged at 4°C, at 15,000 rpm for 15min. Supernatant was taken and stored at -80°C.

### 2.3 iTRAQ labeling and strong cation-exchange fractionation

One hundred milligram of brain tissue was pulverized in liquid nitrogen. Tissue proteins were then isolated using lysate buffer and radioimmunoprecipitation (RIPA). The suspension was sonicated 10 times at 20 watts and centrifuged at 12000 rpm for 20 min at 4°C.

One hundred microgram of protein was digested with trypsin (trypsin to protein ratio 1:100) at 37°C for 4 hours and centrifuged at 12000 rpm. After centrifugation, the protein was solubilized using 0.5 M tetraethylammonium bromide and labeled with 6-plex iTRAQ reagent. Robust cation exchange chromatography was analyzed using a Prominence Nexera UHPLC LC-30A System. Four milliliters of the iTRAQ-labeled peptide mixture were added to buffer A (25 mM NaH2PO4 in 25% ACN, pH 2.7) and transferred to a 4.6×250 mm Ultremex substantial cation exchange column (Phenomenex) and eluted with different concentrations. The elution flow rate was set at 2 mL/min. The elution process is monitored using 214 nm of UV light.

### 2.4 Liquid chromatography tandem-mass spectrometry analysis

Three brain samples per animal (3 × 5 samples from the experimental animals and 3 samples from the control) were obtained and subjected to LC-MS/MS. The mixture of iTRAQ-labeled peptides was resuspended in buffer A (5% Acetonitrile, 0.1% Formic acid), and the concentration of the peptides was adjusted to 0.5 μg/μL. Approximately ten microliter of the resuspension was added *via* autosampler to a 2 cm UHPLC LC-30A System. The assay samples were loaded at 8 μL/min for 4min and then analyzed with a gradient concentration of buffer B (90% Acetonitrile, 0.2% Formic acid) at a rate of 250 NL/min for 35 min. The gradient elution program using buffer B was as follows: 0%-5% for 5min, 5%-15% for 10min, 15%-35% for 60min, 35%-80% for 45min. The peptide fragments were detected at 214 nm of absorbance during the elution process.

Data acquisition and analysis were performed with the TripleTOF 6600 system (AB SCIEX, Concord, ON) and the Nanospray III (AB SCIEX, Concord, ON). Peptide data was obtained with an iron spray voltage of 2.5 kV, a dry gas pressure of 30 psi, a spray gas pressure of 15 psi, and a surface temperature of 150°C. The mass spectrometry was performed at a resolution of over 30,000 FWHM with a time-of-flight mass spectrometry (TOFMS).

### 2.5 Bioinformatic analysis

Differential protein analysis was performed by searching the Uniprot database with Mascot software. The quantification of differentially expressed proteins (DEPs) was calculated using the algorithm included in the Mascot software. The criteria for differential proteins screening were p-value < 0.05 and multiple of difference more than 1.5 times.

GO and KEGG analysis of differential proteins was performed using R language package (Goseq) and KO-BAS3.0 to understand the biological activities involving proteins with significant alteration of expression (Chloe et al., 2022). The software employs authoritative databases in bioinformatic research to create gene symbols, functional annotation of proteins, and pathway enrichment information.

We evaluated the mRNA levels of DEPs using a combined proteomic and transcriptomic approach. The transcriptome data is previously published and available on NCBI (NCBI accession no: PRJNA760987) (Yuan et al., 2022).

### 2.6 Western blotting and immunohistochemistry staining

The iTRAQ data was validated using western blot. Four upregulated (REEP3, REEP4, TEP1, and EEPD1) and one downregulated (GRB2) DEPs were chosen for validation. The protein samples were separated on a 10% SDS-PAGE at 120 volt and transferred to a nylon membrane at 45 mA for 120 minutes. The proteins were incubated with primary antibodies including Rabbit anti-REEP3 (Invitrogen, 1:150), Rabbit anti-REEP4 (Invitrogen, 1:150), Rabbit anti-TEP1 (Invitrogen, 1:150), Rabbit anti-EEPD1 (Invitrogen, 1:150), Rabbit anti-GRB2 (Invitrogen, 1:150). HRP-conjugated goat anti-rabbit IgG was used as a secondary antibody at 1:500. The membrane was visualized using a diaminobenzidine (DAB) substrate solution, and the image was analyzed using western blot detection system.

In addition, immunohistochemistry was performed to confirm the iTRAQ data. Tissue was fixed in paraffin and sectioned to 4 μm for IHC examination. After incubation with methanol followed by 0.75% of hydrogen peroxide, sections were incubated with primary antibody at 4°C overnight, secondary antibody at 37°C for 40 min. Anti-body probing followed the same procedure as shown in western blotting.

### 2.7 Immune infiltration analysis

TIMER 2.0 (http://timer.cistrome.org) is a web platform used for systemic analysis of immune infiltrates in various cancer types. ssGSEA (single-sample gene set enrichment analysis) calculates the enrichment score which represents the degree of up or downregulation of genes in a particular gene set. Taking advantage of the ssGSEA and TIMER, we investigated the association between the protein expression patterns of REEP3, REEP4, TEP1, and EEPD1 and immune infiltration in GBM.

### 2.8 Protein-protein interaction network analysis

The proteins related to RREP3, REEP4, TEP1 and EEPD1 were selected using the STRING (http://string-db.org) database, and the degree of correlation among the proteins was quantified by the Pearson’s correlation coefficient. We used Cytoscape software to build a protein PPI network for visualization. The connection (edge) between nodes represents the protein-protein interaction. The degree of protein correlation was sorted according to the Pearson’s correlation coefficient from large to small, and the top 100 proteins with the highest correlation with RREP3, REEP4, TEP1 and EEPD1 proteins were screened out.

### 2.9 Cancer database analysis

To better understand the underlying mechanisms of carcinogenesis, The Cancer Genome Atlas (TCGA) database was utilized to annotate cancer-related genes and proteins, including mutations, copy number variants, mRNA expression(Tomczak et al., 2015).

We downloaded the RNA-seq data of 23 tumor projects from the TCGA database (https://portal.gdc.cancer.gov) and extracted the data in TPM format. The total number of samples collected was 11124 (Tumor =7260, Normal =3864). We used the R language package (ggplot2 [3.3.6], stats [4.2.1], car [3.1-0]) for statistical analysis and data visualization.

The RNA-Seq data (TPM) of 1846 GBM clinical cases was downloaded from the TCGA database. R language is used for data analysis and processing. Using GEPIA (Gene Expression Profiling Interactive Analysis) dataset (http://gepia.cancer-pku.cn/), we compared the transcript and protein levels in normal brain tissue and GBM tumor in 370 samples (Tang et al., 2017).

## 3 Results

### 3.1 Confirmation of *T. gondii* infection

Sixty days post *T. gondii* infection, the experimental group of masked palm civets began to show clinical signs including loss of appetite and lethargy, the control group remained in good health. The clinical symptoms lasted for 30 days. Ninety days post infection, masked palm civets in experimental and control groups were euthanized and dissected. Brain tissues were removed and stored at -80°C. A 194 bp fragment of B1 gene and a 529bp repeated sequence gene were successfully amplified by PCR, confirming the presence of *T. gondii* tissue cysts in brain tissues.

### 3.2 Differential protein preliminary analysis

We used the iTRAQ-based quantitative proteomic method to identify the proteins and their expression levels in the brain of civet cats infected with *T. gondii*. Of the 377,063 total spectra, 252,916 were identified, among which 14,497 were matched to existing peptides in the database and 5447 were unique peptides (**Supplementary Figure 1A**). A total of 268 DEPs were detected and screened in the brain tissues of masked palm civets, of which 111 were up-regulated, and 157 were down-regulated (**Supplementary Figure 1B**). The differential proteins volcano plot (**Supplementary Figure 1C**) showed the upregulated and downregulated DEPs. As shown in SDS-PAGE, differences in the amount and variety of total, isolated proteins were not observed (**Supplementary Figure 1D**).

### 3.3 Bioinformatics analysis

To identify the biological functions and regulatory pathways the differentially expressed proteins are enriched in, Gene Ontology (GO) and Kyoto Encyclopedia of Genes and Genomes (KEGG) analyses were carried out using R language package (Goseq) and KO-BAS3.0.

**Figure 1:**
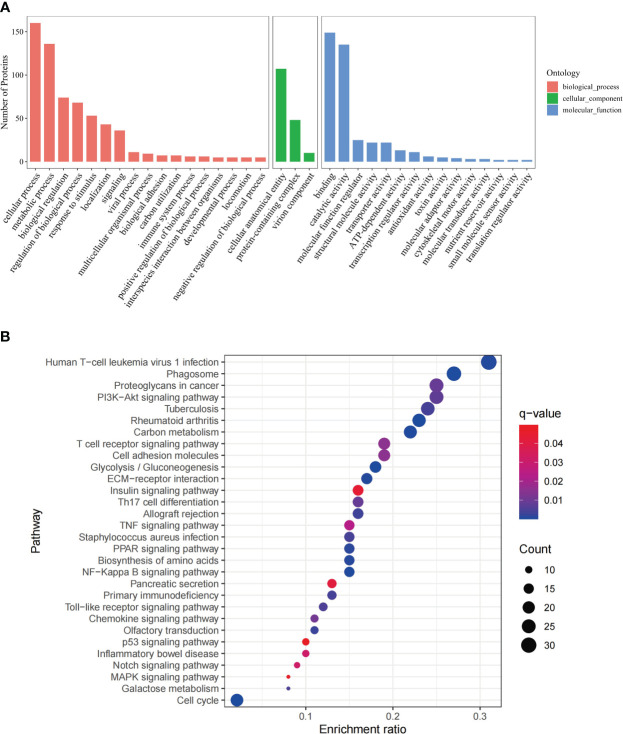
GO annotation and KEGG pathway analysis of DEPs. **(A)** GO analysis of the DEPs, including three categories, biological process, cellular component and molecular function. The x-axis represents the DEPs annotation terms, and the y-axis represents the number of DEPs; **(B)** Bubble chart of KEGG pathway analysis showing the top 30 enriched pathways. The x-axis represents DEPs enrichment ratio, and the y-axis is the KEGG pathway. Enrichment ratio is calculated as the ratio of the number of DEGs to the total number of annotated genes in this pathway. The size of the bubble correlates with the number of DEG annotated in the pathway. q-value (false discovery rate) ranges from 0.01 to 0.04.

GO analysis was based on three categories, namely biological process, cellular component, and molecular function (**Figure 1A**). The 268 DEPs were mapped to 30 pathways *via* KEGG enrichment analysis (**Figure 1B**). In addition, The GO (**Supplementary Figure 2**) and pathway (**Supplementary Figure 3**) analysis were conducted separately on the 111 upregulated and 157 downregulated DEPs.

These include immune regulatory and antitumor pathways, namely, NF-Kappa B signaling pathway, PPAR signaling pathway, Th17 cell differentiation, chemokine signaling pathway, TNF signaling pathway, Notch signaling pathway (Bazzoni and Bentivegna, 2019), MAPK signaling pathway(Lee et al., 2020), p53 signaling pathway (Marei et al., 2021), PI3K-Akt signaling pathway(Tewari et al., 2022), T cell receptor signaling pathway, Toll-like receptor signaling pathway.

Based on log2 fold change, the top five of the upregulated (REEP3, REEP4, TEP1, EEPD1, MYD88) and downregulated (CTLA4, PTPRC, CXCR4, CX3CR1, and GRB2) proteins were compiled in **Table 1**.

**Table 1:** The top five of the upregulated and downregulated DEPs in the brains of *T. gondii* infected masked civets.

Protein	Description	Log_2_(FC)	Differential Expression
REEP3	Receptor accessory protein 3	8.46577	Up
REEP4	Receptor accessory protein 4	6.17868	Up
TEP1	Telomerase associated protein 1	5.61362	Up
EEPD1	Endonuclease/exonuclease/phosphatase family domain containing 1	5.43531	Up
MYD88	Myeloid differentiation primary response gene 88	3.45467	Up
CTLA4	Cytotoxic T-lymphocyte-associated protein 4	-1.11099	Down
PTPRC	Protein tyrosine phosphate receptor type C	-2.09878	Down
CXCR4	C-X-C motif chemokine receptor 4	-4.67890	Down
CX3CR1	C-X3-C motif chemokine receptor 1	-5.19975	Down
GRB2	Growth factor receptor-bound protein 2	-7.68899	Down

### 3.4 Western blot

**Figure 2:**
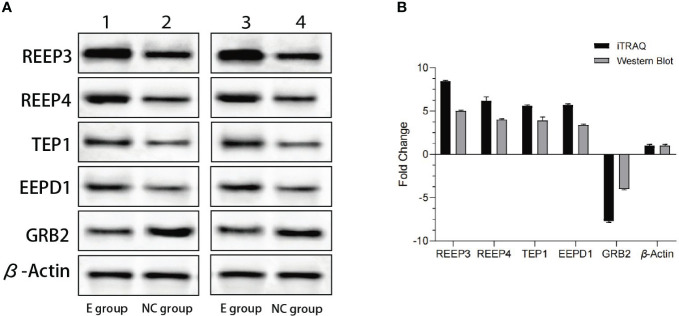
Western blot analysis of the expression levels of REEP3, REEP4, TEP1, EEPD1 and GRB2. **(A)** Lane 1 shows the data from the experimental group. Lane 2 shows the data from the control group. Lane 3 and lane 4 are data from a biological replicate. β-Actin is the endogenous control; **(B)** Quantification of the results shown in panel A using Gel-Pro Analyzer 4.0 software. No significant difference (p = 0.1) was observed comparing data from western blot and iTRAQ.

To validate the iTRAQ data, we performed western blot. The top four most significantly upregulated proteins (REEP3, REEP4, TEP1 and EEPD1) and one significantly downregulated protein (GRB2) were selected (**Table 1**). As shown in **Figure 2**, REEP3, REEP4, TEP1, and EEPD1 were upregulated, while GRB2 was downregulated, consistent with the iTRAQ data.

### 3.5 Proteomic and transcriptomic analyses of the infected brain tissue

**Figure 3:**
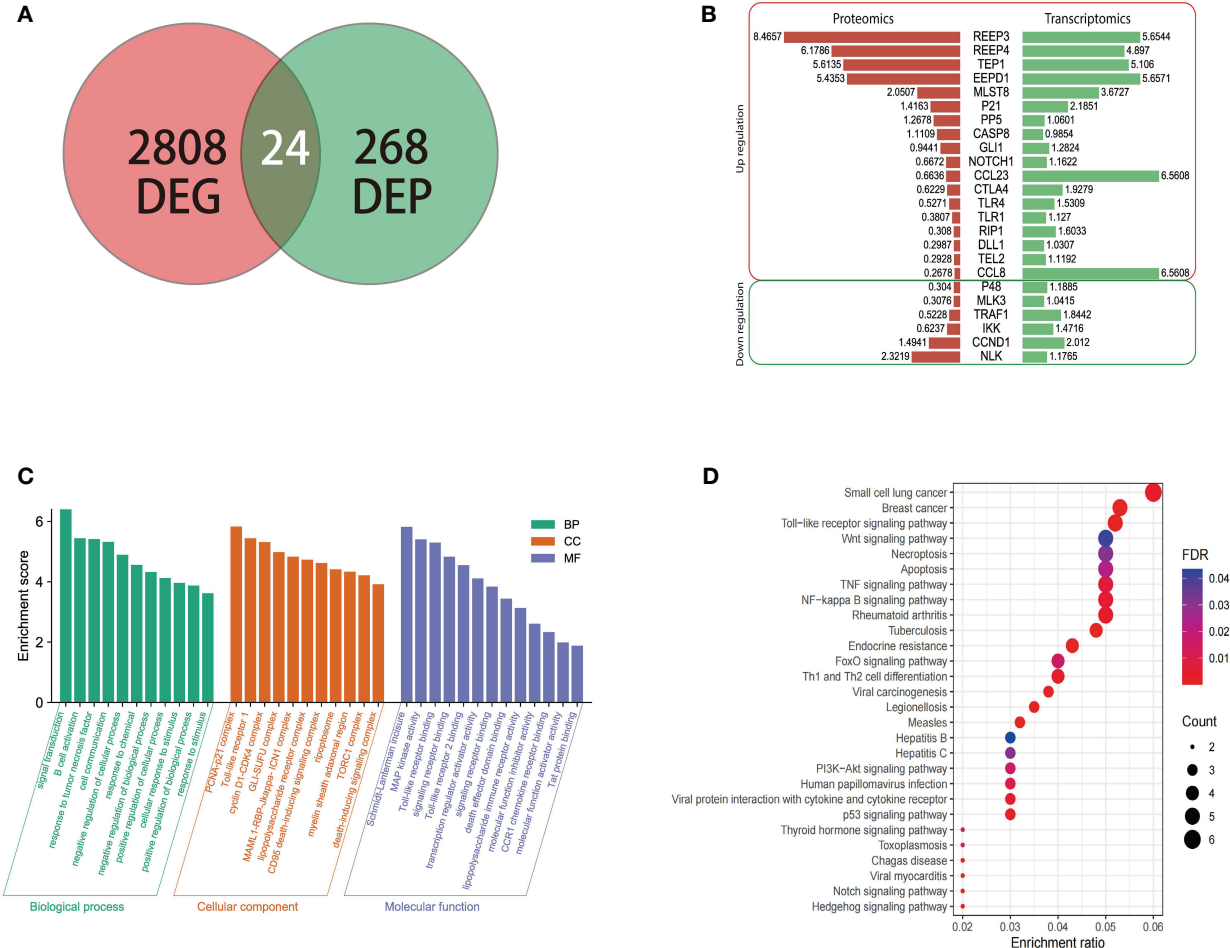
The transcriptomic, proteomic analysis of brain tissue of masked civet infected with *T. gondii*; **(A)** Identification of 24 genes that were differentially expressed on both mRNA and protein levels. Proteomic data from this study was combined with the transcriptomic data from our previous study; **(B)** Gene regulation patterns of the 24 DEGs. The numbers are the log2 (fold change) of up or down regulation; **(C)** The GO functional annotation of the 24 DEGs in terms of biological process, cellular component, and molecular function. **(D)** KEGG pathway enrichment analysis showed that the 24 DEPs were mapped to 28 pathways. The red to purple color gradient shows the FDR (false discovery rate), and the size of the circle represents the number of proteins.

To understand the expression of different proteins in the brain of masked palm civet infected with *T. gondii* at the transcriptional and protein levels, we performed proteomics and transcriptomics. Among the 268 differentially expressed proteins and 2808 differentially expressed genes, twenty-four showed consistent results (**Figure 3A**). The regulation patterns of these 24 genes were shown in **Figure 3B**. Despite variations in the expression amount, the two omics showed consistent expression patterns of these genes (**Figure 3B**). Noteworthily, REEP3, REEP4, TEP1, and EEPD1 were upregulated in both methods.

The twenty-four genes of interest were therefore subjected to functional annotation in categories of biological process, cellular component, and molecular function (**Figure 3C**). Gene Ontology analysis showed that in the biological process category, these 24 genes were involved in signal transduction, B cell activation, response to tumor necrosis factor, and other similar processes. These genes were enriched in cellular components such as PCNA-p21 complex, Toll-like receptor 1, and cyclin D1-CDK4 complex (**Figure 3C**). In terms of molecular function, these genes were involved in Schmidt-Lanterman incisure, MAP kinase activity, Toll-like receptor binding, among others (**Figure 3C**).

KEGG pathway enrichment analysis showed that the 24 genes were involved in pathways such as small-cell lung cancer, breast cancer, Toll-like receptor signaling pathway (**Figure 3D**). In addition to the cancer pathways identified, the 24 genes were mapped to the following signaling pathways, namely PI3K-Akt, Wnt, P53, Notch, and NF - Kappa B signaling pathway which were shown to be the critical pathways (Hellmark and Segelmark, 2014; Xie et al., 2019).

### 3.6 Immunohistochemistry analysis of the brain tissues of infected animals

**Figure 4:**
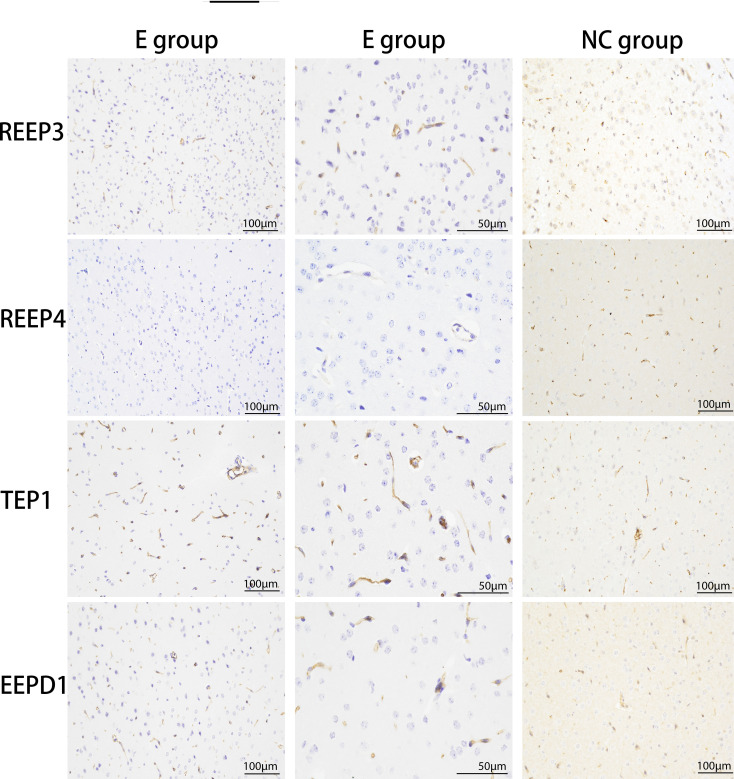
The upregulation of REEP3, REEP4, TEP1 and EEPD1 in the brain of masked palm civets infected with *T. gondii*. E group: experimental group (scale bar, 100 μm or 50 μm). NC group: control group (scale bar, 100 μm). The second column of the experimental group showed the data in a bigger magnification. Upon hematoxylin staining, blue color indicates the expression of REEP3, REEP4, TEP1, and EEPD1 proteins in the brain of masked palm civets infected with *T. gondii*.

We used immunohistochemistry to verify the expression levels of REEP3, REEP4, TEP1, and EEPD1 in the brain of masked palm civets infected with *T. gondii*. The results showed that the expression of REEP3, REEP4, TEP1, and EEPD1 proteins were higher in *T. gondii* infected brain tissue than normal tissue (**Figure 4**). This finding confirmed that *T. gondii* infection altered the protein expression of REEP3, REEP4, TEP1, and EEPD1 in the brain of masked palm civets.

### 3.7 Immune infiltration correlation analysis

**Figure 5:**
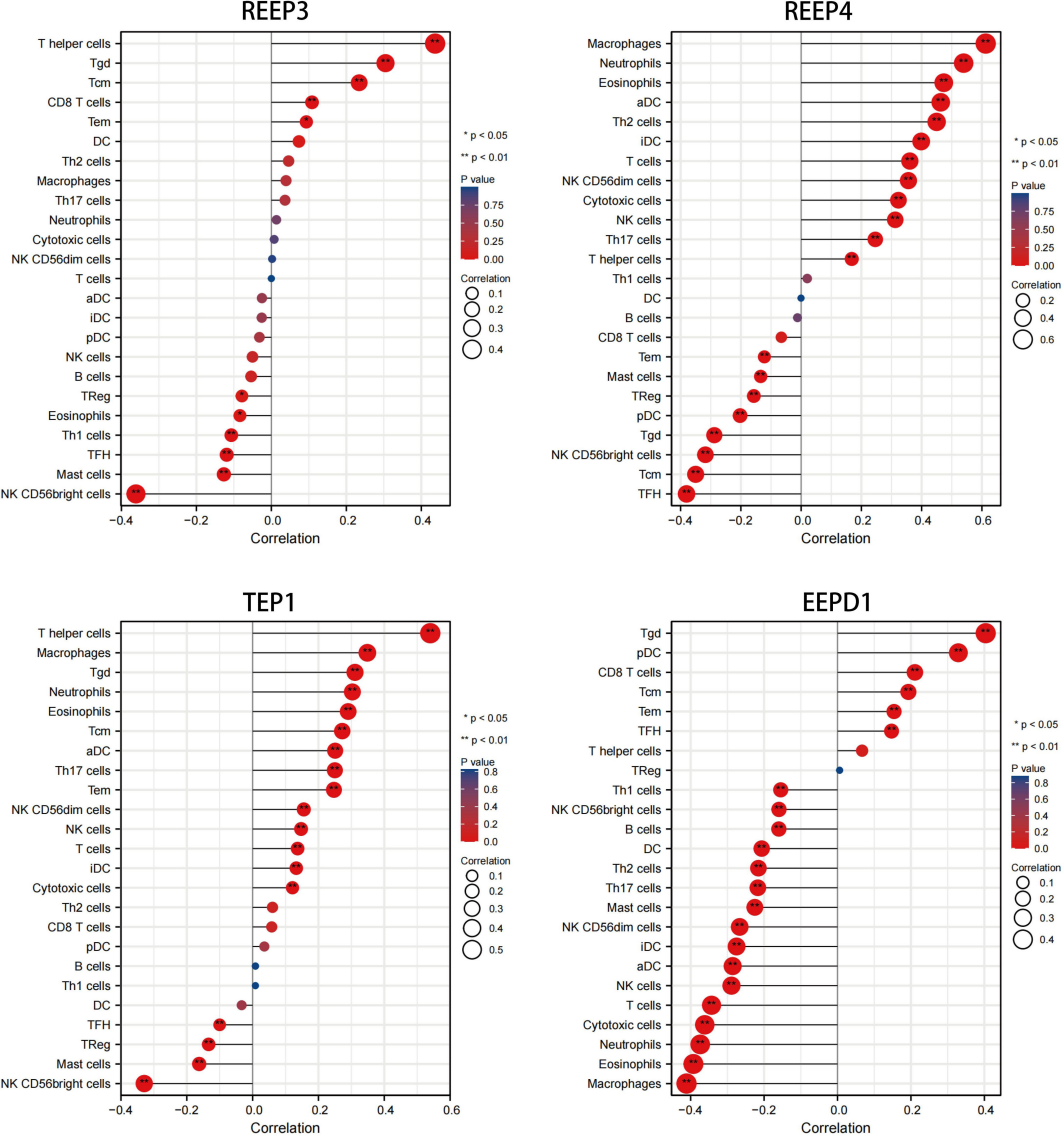
Correlation analysis of REEP3, REEP4, TEP1 and EEPD1 expression immune infiltration. The x-axis represents the correlation index. A negative number indicates a negative correlation between the protein and immune cell, a positive number indicates a positive correlation. The y-axis represents the immune cells. The circle size shows the correlation degree with a bigger circle representing higher correlation. A red to blue color gradient indicates p-value (*p < 0.05, **p < 0.01).

We used immune infiltration analysis to identify respective immune cells that were associated with the expression regulation of REEP3, REEP4, TEP1, and EEPD1. Data was analyzed using TIMER 2.0 databases (Li et al., 2017; Ru et al., 2019). The immune cells that were associated with the up and down regulation of these four proteins were shown in **Figure 5**. The upregulation of REEP3 was associated with T helper cell, T gamma delta (Tgd) cell, and T central memory (Tcm) cell. The downregulation of REEP3 was linked to CD56bright NK cell, Mast cell, and T follicular helper (Tfh) cell. The top three immune cells that were associated with REEP4 upregulation included macrophage, neutrophil, and eosinophil. In the case of TEP1, the T helper cell, macrophage, and Tgd were as-sociated with its upregulation. Tgd cell, plasmacytoid DC (pDC) cell, and CD8 T cell were related to the upregulation of EEPD1. Our finding suggests that the elevated expression of REEP3, REEP4, TEP1, and EEPD1 modulates immune cell infiltration in the brain microenvironment.

### 3.8 Protein-protein interaction analysis and pathway prediction

**Figure 6:**
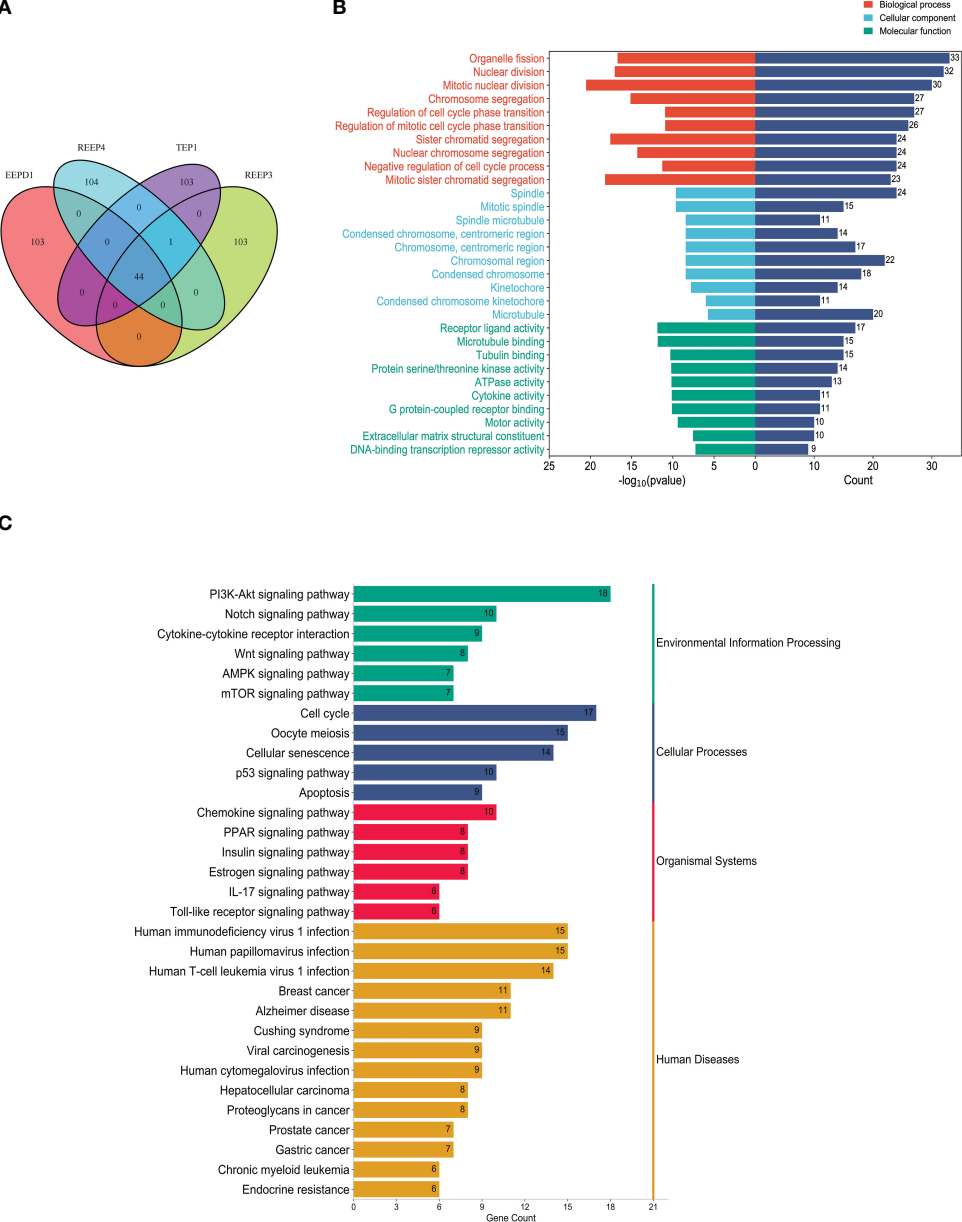
Protein-protein interaction analysis of hub proteins (REEP3, REEP4, TEP1 and EEPD1). **(A)** Proximity protein correlation analysis. REEP3 was connected to 103 proteins, REEP4 was connected to 104 proteins, TEP1 was connected to 103 proteins, and EEPD1 was also connected to 103 proteins. A total of 44 proteins were co-expressed in REEP3, REEP3, TEP1 and EEPD1; **(B)** GO annotation of the 44 proteins. The x-axis represents the protein count and -log10 (p-value), the y-axis represents the protein annotation term; **(C)** KEGG pathway enrichment analysis. The 44 DEPs were enriched in 31 pathways. The x-axis represents protein count, and the y-axis shows the KEGG pathways.

To better understand the regulatory mechanism of GBM, we examined the neighboring proteins that were closely associated with REEP3, REEP4, TEP1 and EEPD1 in the protein networks. As shown in **Supplementary Figure 4**, the STRING database was utilized to screen the respective, top 100 proteins with close relation to REEP3, REEP4, TEP1, and EEPD1. The four groups of related proteins were analyzed collectively and a total of 44 proteins were found to be linked to all four proteins (**Figure 6A**). GO annotation provided functional annotation for the 44 proteins regarding biological processes, cellular components, and molecular functions (**Figure 6B**). The 44 proteins were mapped to the biological processes including cell division, chromatid segregation, and cell cycle phase transition. These proteins were implicated in cellular components such as spindle, microtubule, and kinetochore, and molecular function of receptor ligand activity, microtubule binding, and tubulin binding.

KEGG enrichment analysis identified the pathways involving the 44 proteins. As shown in **Figure 6C**, the majority of these proteins were significantly enriched in immunoregulation and cancer pathways. These included PI3K-Akt signaling pathway, cytokine-cytokine receptor interaction, Wnt signaling pathway, AMPK signaling pathway, breast cancer, prostate cancer, gastric cancer. This confirmed that REEP3, REEP4, TEP1, and EEPD1 and their network proteins were involved in immunoregulatory functions and cancer development.

### 3.9 Assessment of REEP3, REEP4, TEP1 and EEPD1 mRNA expression in pan-cancer analysis

**Figure 7:**
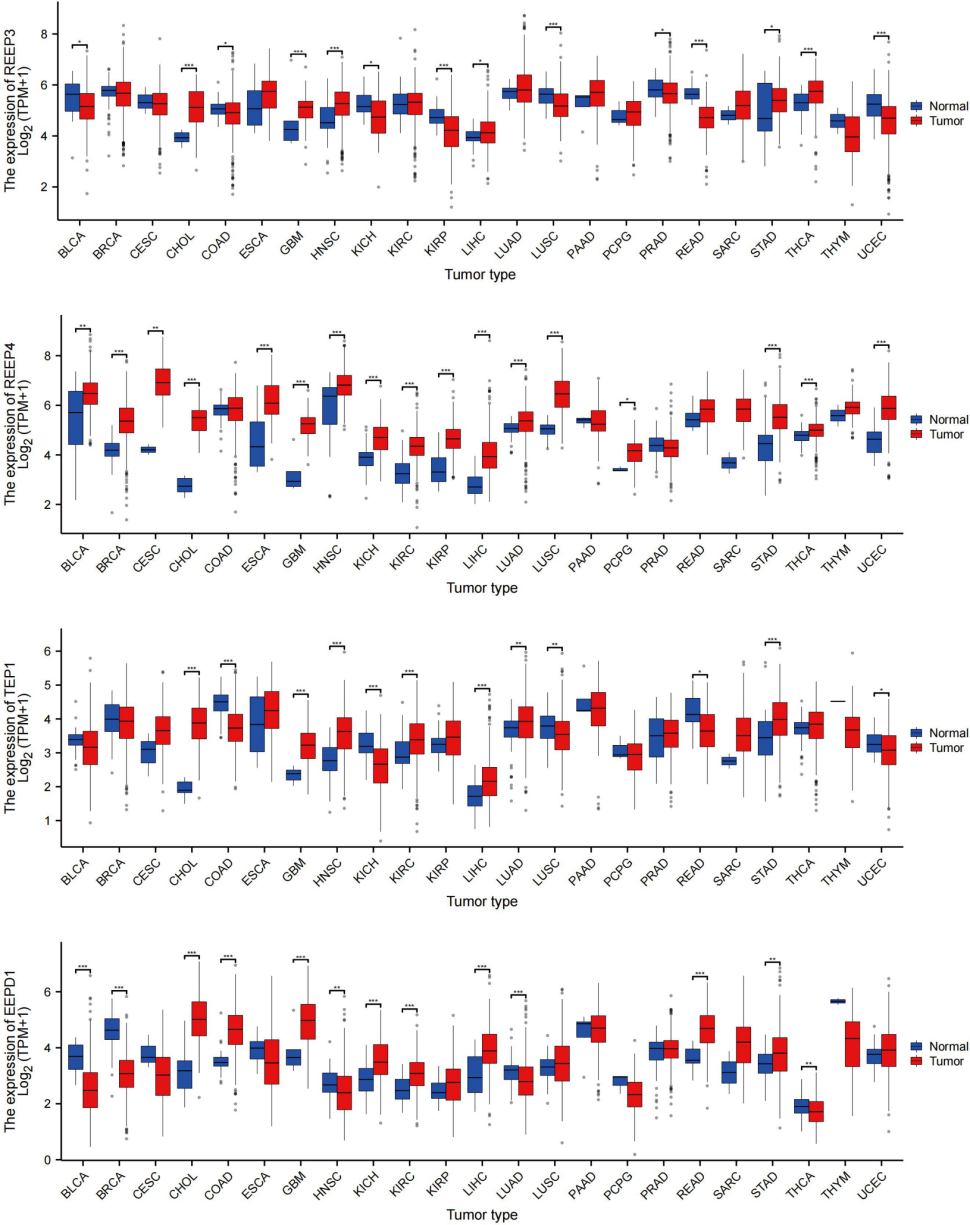
REEP3, REEP4, TEP1 and EEPD1 TCGA Pan-Cancer Analysis. The mRNA expression levels of 11124 samples (Tumor =7260, Normal = 3864) were obtained from the TCGA database. The expression amounts of REEP3, REEP4, TEP1, and EEPD1 in the tumor versus normal tissue samples were plotted. Significance analysis used t-test (*p < 0.05, **p < 0.01, ***p < 0.001).

To evaluate REEP3, REEP4, TEP1 and EEPD1 mRNA levels in multiple tumor and normal samples, we downloaded and compiled data from 11124 samples (Tumor =7260, Normal =3864) from the TCGA database. As shown in **Figure 7**, REEP3, REEP4, TEP1 and EEPD1 was differentially expressed in 14, 17, 14, 13 tumor types, respectively. REEP4 was upregulated in all 17 types of tumor as compared to normal tissue while the expression level of the rest of the efforts is tumor-specific. All four effectors were upregulated in CHOL (cholangiocarcinoma), GBM (glioblastoma), LIHC (liver hepatocellular carcinoma), and STAD (stomach adenocarcinoma).

Taking advantage of Gene Expression Profiling Interactive Analysis (GEPIA) and The Cancer Genome Atlas Program (TCGA), we compared mRNA expression of REEP3, REEP4, TEP1, and EEPD1 between GBM tumor and normal brain tissue (**Supplementary Figure 5A**). The result showed that the expression of REEP3, REEP4, TEP1, and EEPD1 was significantly higher in GBM tumor tissue (**Supplementary Figure 5A**).

Next, we obtained 1846 GBM samples from the TCGA database. Of which, 1157 samples were from normal brain tissues and 689 were from GBM tumor tissues. As shown in (**Supplementary Figure 5B**), the REEP3, REEP4, TEP1, and EEPD1 had significantly higher expression in GBM tumor tissues compared to normal tissues.

## 4 Discussion

In this study, we investigated the proteome of the brain of *T. gondii* infected masked palm civets. Consistent with our previous transcriptomic data, we found that the DEPs were enriched in immune regulatory pathways including Toll-like receptor (TLR), NF-Kappa B, T cell receptor, Chemokine, and PI3K-Akt signaling pathways. The top four most upregulated DEPs were REEP3, REEP4, TEP1, and EEPD, which were also upregulated on transcriptional level.

Receptor expression-enhancing proteins (REEPs) are a family of conserved proteins that is critical to many physiological processes such as morphogenesis and remodeling of endoplasmic reticulum (ER). In addition to the functions related to ER and microtubule skeleton, REEPs contribute to disease development (Fan et al., 2022). REEP3 and REEP4 are abundant in the brain. REEP3 is associated with depression, Alzheimer’s disease, obsessive-compulsive disorder, and autism (Fan et al., 2022). The disruption of REEP3 expression due to a position effect could lead to autism (Castermans et al., 2007). REEP3 is a prognostic marker in liver cancer (Wei et al., 2020). REEP4 mutations were associated with neurological disorders such as Meige syndrome and blepharospasm (Fan et al., 2022). A study found that in pancreatic cancer, high expression of REEP4 was associated with tumor invasion and poor prognosis (Giardiello et al., 2020).

Telomerase-associated protein 1(TEP1) is a constitute of ribonucleoprotein complex which is responsible for the activity of telomerase (Duan et al., 2021). In a Drosophila glioma model, the downregulation of TEP1 reduced the activity of Yki and curtailed the growth of glioma (Gangwani et al., 2020). Mutations in TEP1 were thought to be responsible for the development of cerebral palsy (Wang et al., 2021). EEPD1 plays a role in DNA damage repair, in which it cleaves the stalled replication fork and thus initiates the homologous recombination repair. EEPD1was thought to be responsible for mitotic catastrophe in breast cancer cells (BRCA1 mutant) in the absence of RAD52, suggesting its critical role in cancer cell survival (Hromas et al., 2017). As a replication stress nuclease, EEPD1 was found to be overexpressed in various malignancies (e.g., brain, breast, kidney, lung) likely owing to its function of helping cancer cells cope with oncogenic stress (e.g., radiation, genotoxins) (Nickoloff et al., 2022). Defect in EEPD1 predisposes cells to cancer due to its role in damage repair (Nickoloff et al., 2022). EEPD1 thus serves as a biomarker for stressed cancer cells and a target of cancer therapeutics (Nickoloff et al., 2022). Taken together, REEP3, REEP4, TEP1, and EEPD1 were related to many cellular functions including cell proliferation, differentiation, the pathogenesis of neurological disorders and cancer biology.

The GEPIA and TCGA datasets were used to analyze the expression patterns of REEP3, REEP4, TEP1, and EEPD1 in tumor tissue and normal tissue. The expression of these four proteins was elevated in many cancer types including GBM. KEGG pathway analysis of the top 155 DEPs revealed pathways that were crucial in dealing with cellular stresses due to *T. gondii* infection. These conserved pathways govern cell proliferation, apoptosis, cell cycle, and differentiation. The aberrant signaling due to mutations or abnormal expression of genes is responsible for oncogenesis. These differentially expressed pathways include the P53 signaling pathway, Notch signaling pathway, and the PI3K-Akt signaling pathway.

The P53 signaling pathway was activated when cells were exposed to genotoxic and cytotoxic stresses, which allowed cells to turn on transcriptional regulation and lead to cell cycle arrest, DNA repair, and apoptosis of tumor cells (Marei et al., 2021). The activation of this pathway offers tumor cells an advantage to cope with environmental stresses such as lack of nutrients, hypoxia, low PH (Tewari et al., 2022). Mutations in TP53, the gene that encodes P53, were found in various human cancers (Mao et al., 2012; Marei et al., 2021). The notch signaling pathway consists of five ligands (Dll1-4, Jagged 1-2) and four receptors (Notch 1-4). Notch signaling pathway is shown to regulate neural stem cells and glioma stem cells (GSCs) and therefore is essential in neurogenesis and carcinogenesis. Alterations in the notch signaling pathway contribute to cancers. In fact, genes that are involved in the notch signaling pathway were found to be upregulated on mRNA and protein levels in GBM (Bazzoni and Bentivegna, 2019), consistent with our finding where Notch1 and Dll1 were upregulated transcriptionally and translationally (**Figure 3B**). KEGG pathway analysis of the 24 DEPs and the 44 network proteins identified the Wnt signaling pathway. Wnt signaling cascade along with RAS/MAPK, Notch, Hedgehog, PI3K/Akt pathways contributed to the stemness in GBM (Latour et al., 2021). WNT cascade was found to be overactive in GBM allowing GSCs (glioblastoma stem cells) to replicate aggressively (Latour et al., 2021).

REEP3 and EEPD1 were positively correlated with T cell infiltration including CD8+ T cells, Tcm, Tgd. REEP4, and TEP1 were positively correlated with the infiltration of macrophage, neutrophils, eosinophile, DC, NK cells among others. The alterations in the expression of these four proteins modulated the infiltration of innate and adaptive immune cells in the brain. Higher expression levels of REEP3, REEP4, EEPD1, and TEP1 were found in oligodendrocyte, monocyte/macrophage, AC-like malignant cells, and monocyte/macrophage, respectively, although it should be noted that the expression levels vary significantly among different datasets.

*T. gondii* infection has been associated with increased risk of glioma development. A study of two cohorts of 360 cases with approximately the same number of matched controls showed that people with glioma were more likely to have *T. gondii* antibodies (Hodge et al., 2021). In another study, data from 2323 brain tumor patients and 5131 healthy controls were included in a meta-analysis. They found that the risk of brain tumor was higher in *T. gondii* infected individuals than those without infection (Abdollahi et al., 2022). Whether *T. gondii* infection mediates the risk of glioblastoma is unknown. However, we found that the four proteins that were upregulated in *T. gondii* infected brains of the masked palm civets were also upregulated in glioblastoma patients. MicroRNA-21 derived from *T. gondii* infected microglial cells promoted the growth of U86 glioma cells through suppressing antitumoral genes (FoxO1, PTEN, and PDCD4) (Jung et al., 2022). In general, *T. gondii* infection in the CNS caused inflammation and the inhibition of apoptosis (Jung et al., 2022). Indeed, we found the upregulation of immune-related effectors and pathways in *T. gondii* infected brain tissue.

We went a step further and investigated whether the expression of the four proteins is associated with GBM outcome. We retrieved and compiled the clinical data from the TCGA database and categorized GBM patients into high and low risk groups based on the expression levels of the four proteins. As shown in **Supplementary Figure 6A**, the 50-month overall survival rate between the high and low expression groups of REEP3 (*P* = 0.001), REEP4 (*P* < 0.001), TEP1 (*P* < 0.001) and EEPD1 (*P* < 0.001) was significantly different in GBM patients. The high expression of REEP3 and EEPD1 was associated with increased the survival time of patients. The high expression of REEP4 and TEP1 was associated with decreased survival time of patients (**Supplementary Figure 6A**).The diagnostic strength of EEP3, REEP4, TEP1, and EEPD1 in GBM was scored by drawing ROC curves with R (Proc) software. As shown in **Supplementary Figure 6B**, the area under the curve (AUC) for REEP3 was 0.795 (CI: 0.774-0.861), REEP4 0.844 (CI: 0.852-0.863), TEP1 0.817 (CI: 0.786-0.848), and EEPD1 0.963 (CI: 0.952-0.973).

## 5 Conclusion

Differentially regulated proteins and signaling pathways in *T. gondii*-infected masked palm civets were identified by omics and bioinformatic methods. These genes and proteins were associated with many physiological processes and cellular signaling pathways including those that are related to immune response and cancer associated.

## Data availability statement

The mass spectrometry proteomics data of this article are available in the iPro X (accessionno. PXD038083, https://www.iprox.cn//page/project.html?id=IPX0005374000). The transcriptomedata generated in this study have been deposited into NIH Gen-Bank (accession no. RJNA760987, https://www.ncbi.nlm.nih.gov/bioproject/?term=+PRJNA760987). The tumor data and control data were sourced from TCGA and GEPIA database.

## Ethics statement

The animal study was approved by Animal Administration Committee of South China Agricultural University approved all animal experiments (No. SCAU2021f163). The study was conducted in accordance with the local legislation and institutional requirements.

## Author contributions

HY: Data curation, Formal Analysis, Investigation, Methodology, Project administration, Resources, Software, Supervision, Validation, Visualization, Writing – original draft, Writing – review & editing. TJ: Data curation, Formal Analysis, Investigation, Methodology, Project administration, Software, Supervision, Writing – original draft, Writing – review & editing. WZ: Conceptualization, Writing – review & editing, Data curation, Investigation, Methodology, Software, Supervision. ZPY: Writing – review & editing, Investigation, Software. SL: Writing – review & editing, Data curation, Methodology, Supervision, Validation. XW: Writing – review & editing, Formal Analysis, Project administration, Resources, Visualization. XZ: Writing – review & editing, Methodology, Project administration, Supervision, Validation. SQ: Writing – review & editing, Methodology, Supervision. YM: Writing – review & editing, Formal Analysis, Methodology, Project administration, Supervision, Validation. XZ: Writing – review & editing, Conceptualization, Data curation, Investigation, Software. Z-GY: Supervision, Validation, Writing – review & editing, Conceptualization, Data curation, Formal Analysis, Investigation, Methodology, Project administration, Software.
