# Supplementary material for: Multiomics and bioinformatics identify differentially expressed effectors in the brain of Toxoplasma gondii infected masked palm civet
Source: Front Cell Infect Microbiol. 2023 Sep 25;13:1267629. doi: 10.3389/fcimb.2023.1267629 (PMC10561248; doi:10.3389/fcimb.2023.1267629)
Supplement: Supplementary file 1 [file DataSheet_1.docx]

Supplementary Material


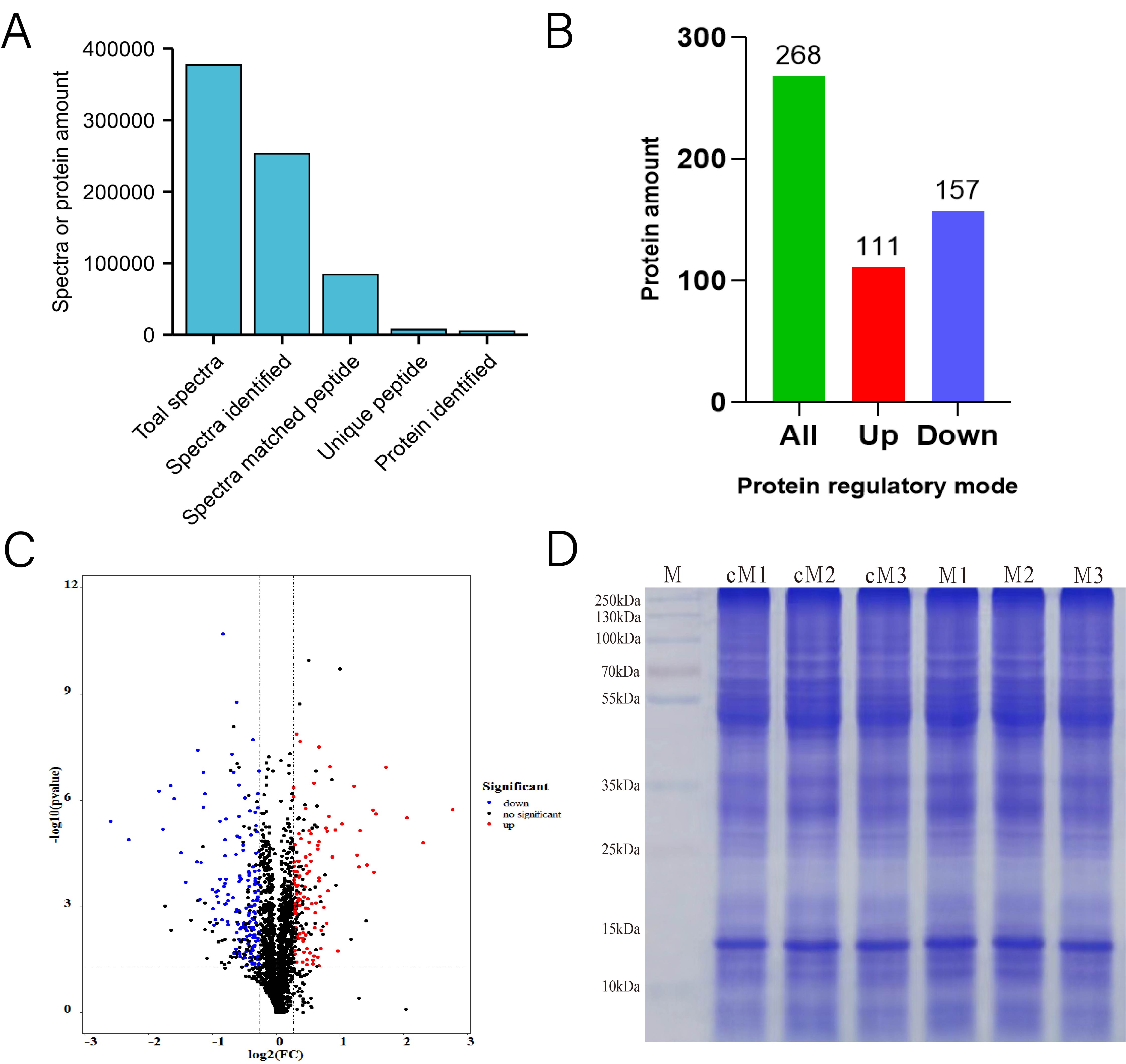


**Supplementary Figure 1.** LC-MS/MS identified DEPs. (A) and (B): bar graphs showing the numbers of identified spectra and the DEPs. (C) Volcano plot of DEPs. Red dots indicate upregulation and blue dots indicate downregulation. The log2 fold change(x-axis) is plotted against the −log10(p-value) (y-axis). The horizontal line shows the threshold of p-value = 0.05. (D) Protein SDS-PAGE showing no differences in the total amount and variety of proteins among samples. M denotes molecular marker. cM1,2,3 denote the three technical replicates in the control group. M1,2,3 are the three technical replicates in the experimental group.


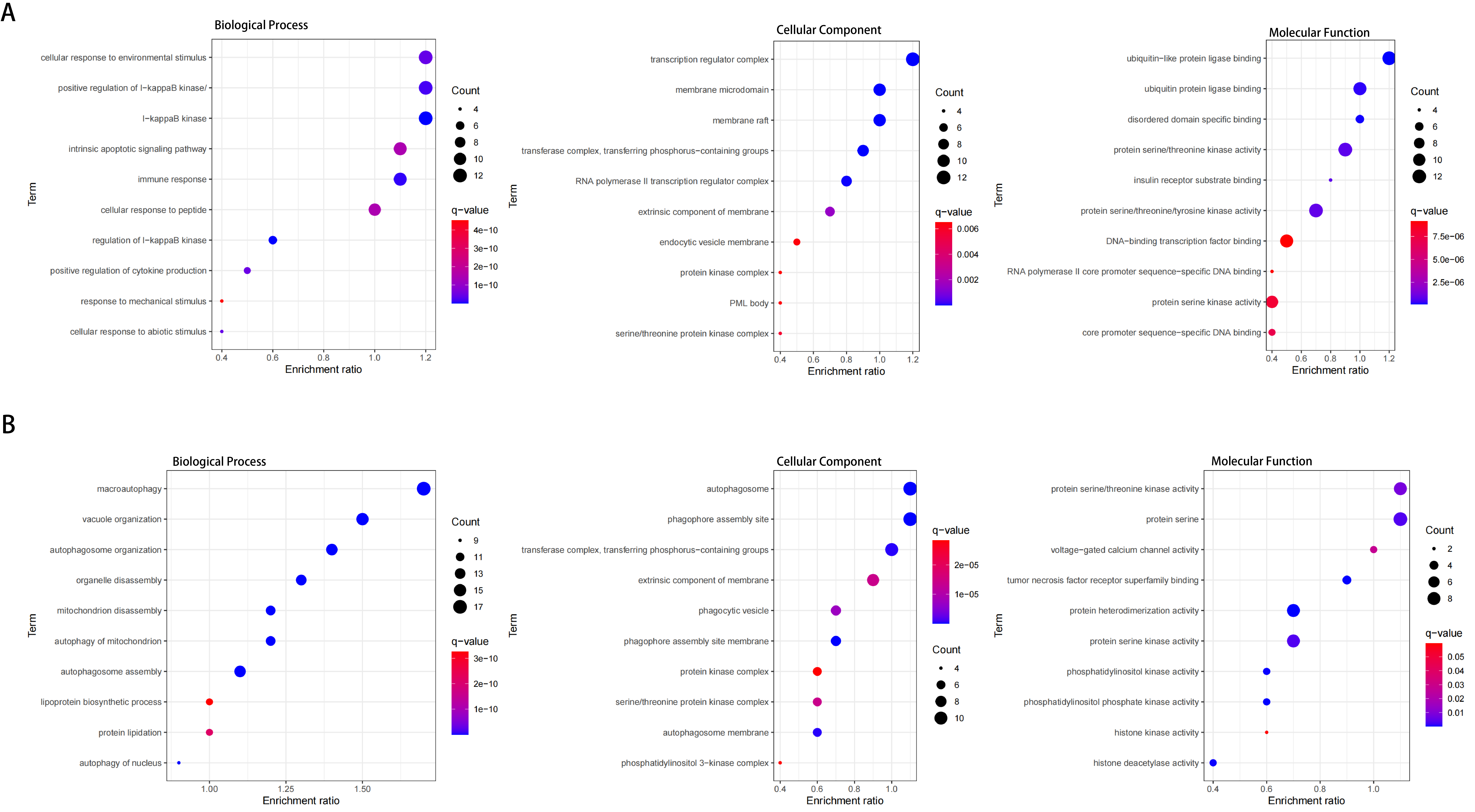


**Supplementary Figure 2.** GO annotation analysis of DEPs. (A) and (B) GO analysis of the upregulated 111 DEPs and downregulated 157 DEPs, respectively, including three categories, biological process, cellular component, and molecular function. The x-axis represents the DEPs annotation terms, and the y-axis represents the number of DEPs. The size of the bubble correlates with the number of DEG annotated in the pathway.


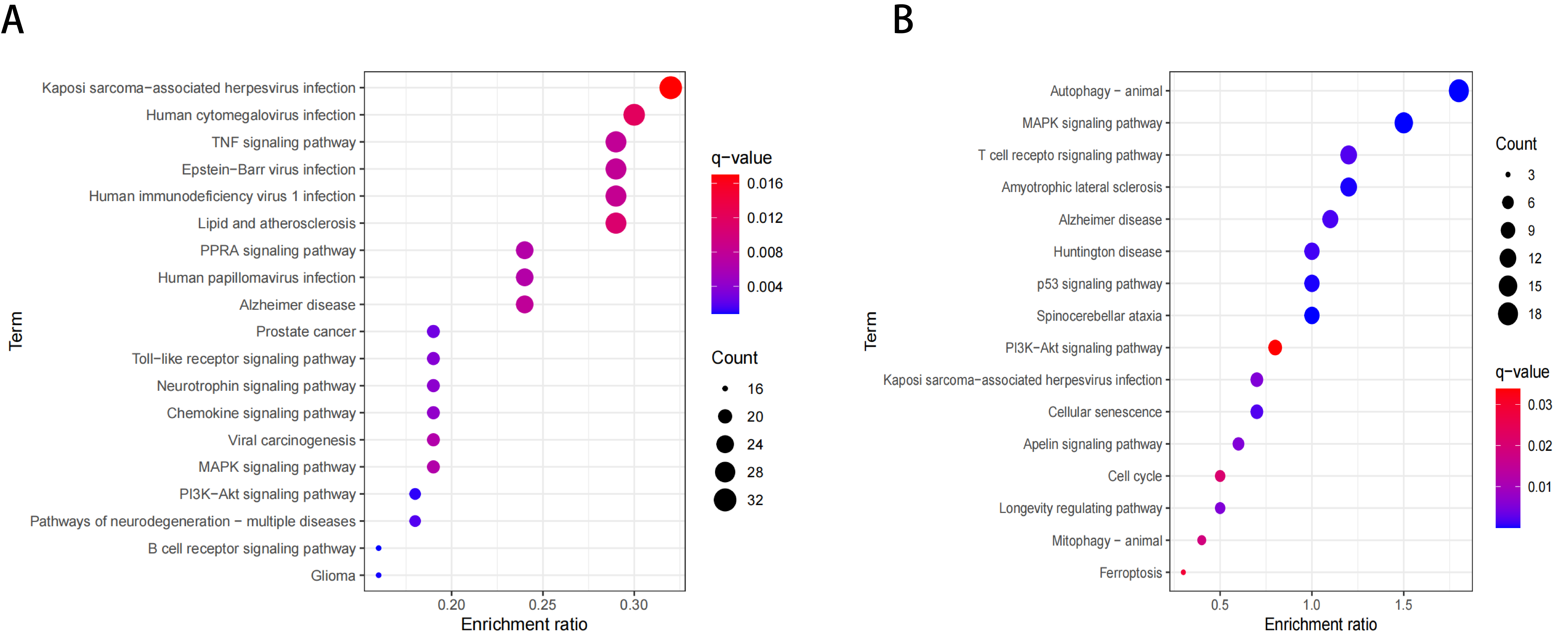


**Supplementary Figure 3.** KEGG pathway analysis of DEPs. (A) and (B) KEGG pathway analysis of the upregulated 111 DEPs and downregulated 157 DEPs, respectively. The x-axis represents DEPs enrichment ratio, and the y-axis is the KEGG pathway. Enrichment ratio is calculated as the ratio of the number of DEGs to the total number of annotated genes in this pathway. The size of the bubble correlates with the number of DEG annotated in the pathway.


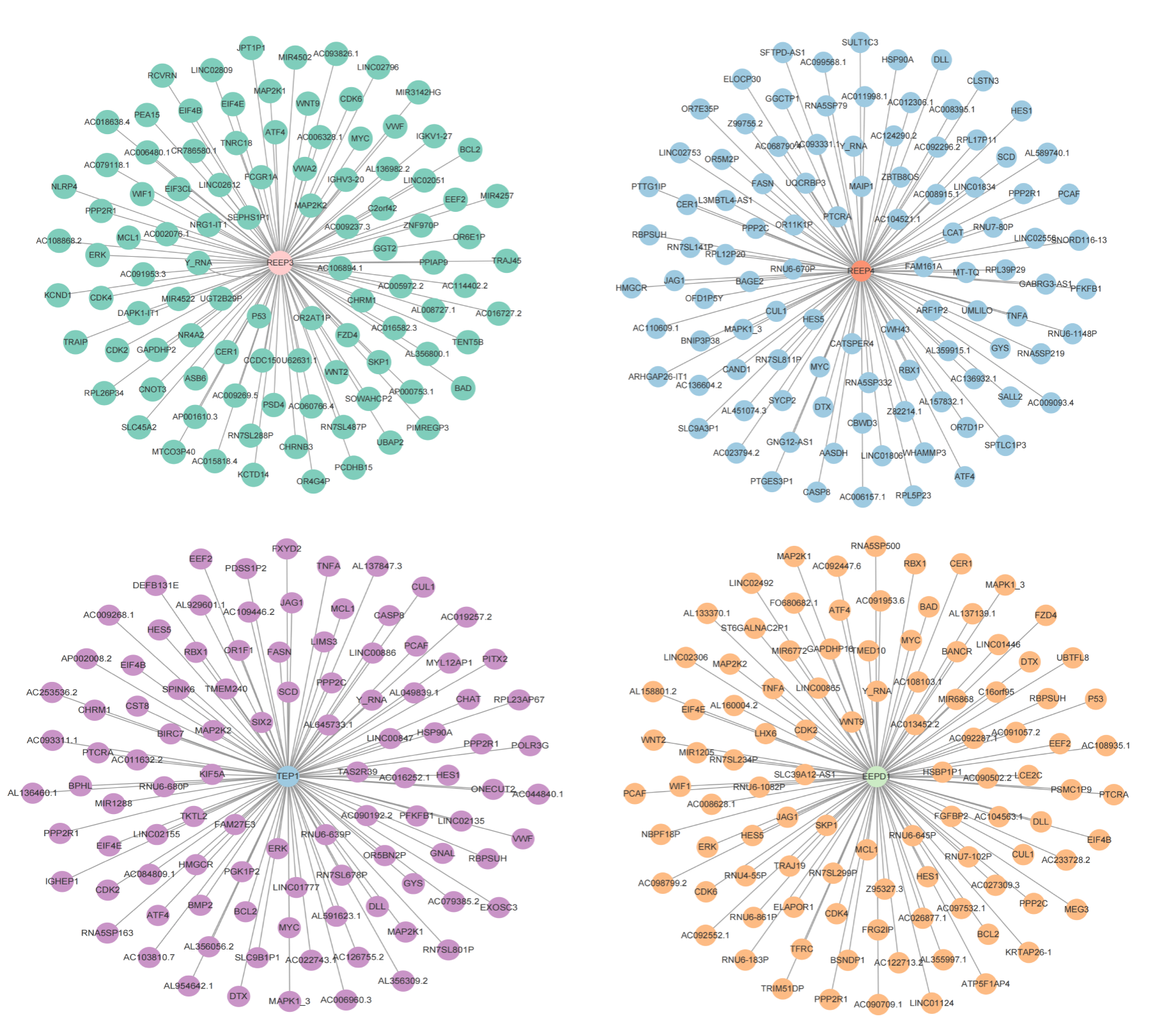


**Supplementary Figure 4.** Protein-Protein interaction network construction. The correlation analysis of REEP3, REEP4, TEP1 and EEPD1 and their respective neighboring proteins.


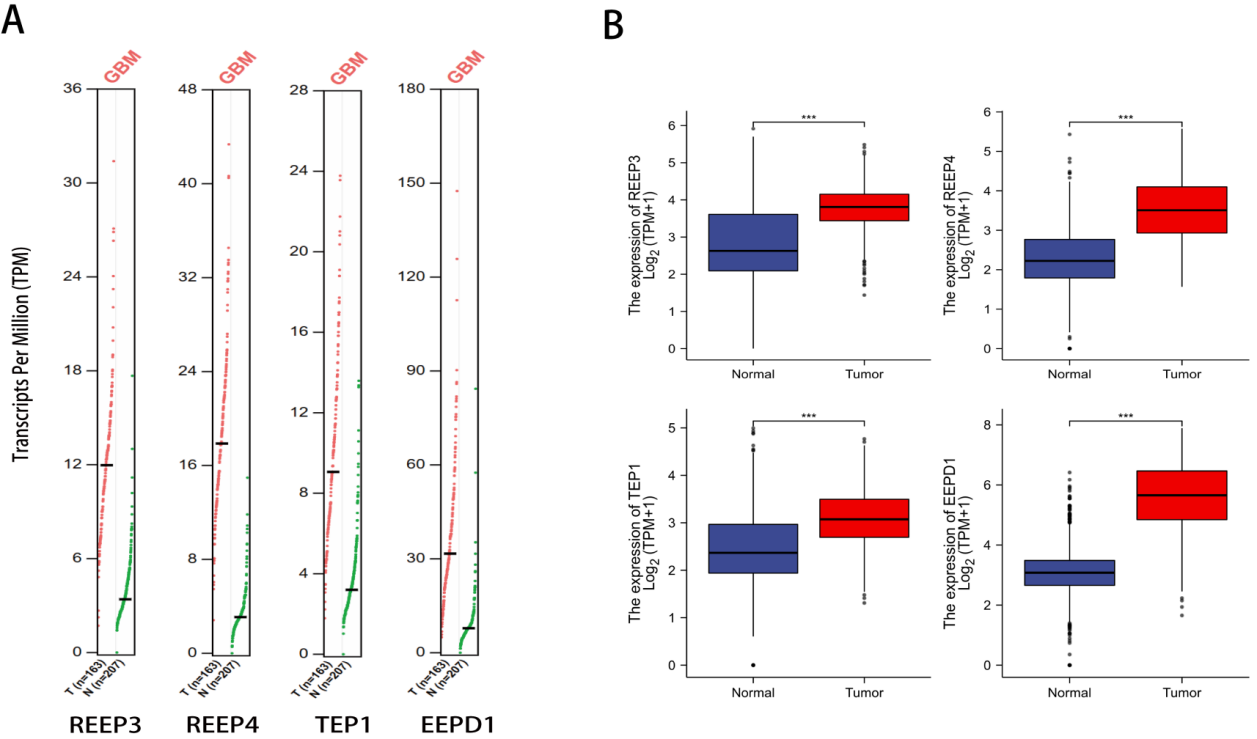


**Supplementary Figure 5.** Analysis of expression levels of REEP3, REEP4, TEP1 and EEPD1 in normal brain versus tumor brain using GEPIA and TCGA databases; (A) The transcript amount in GBM versus normal brain. The 370 samples were obtained from the GESPA database. The GBM group was shown in red (n=163) and the control group (n=207) was shown in green; (B) The mRNA expression levels of the 1846 samples (GBM: 689, normal: 1157) obtained from the TCGA database. The expression amounts of REEP3, REEP4, TEP1, and EEPD1 in the tumor versus normal brain were plotted. Significance analysis used t-test (*** *p* ≤ 0.001).


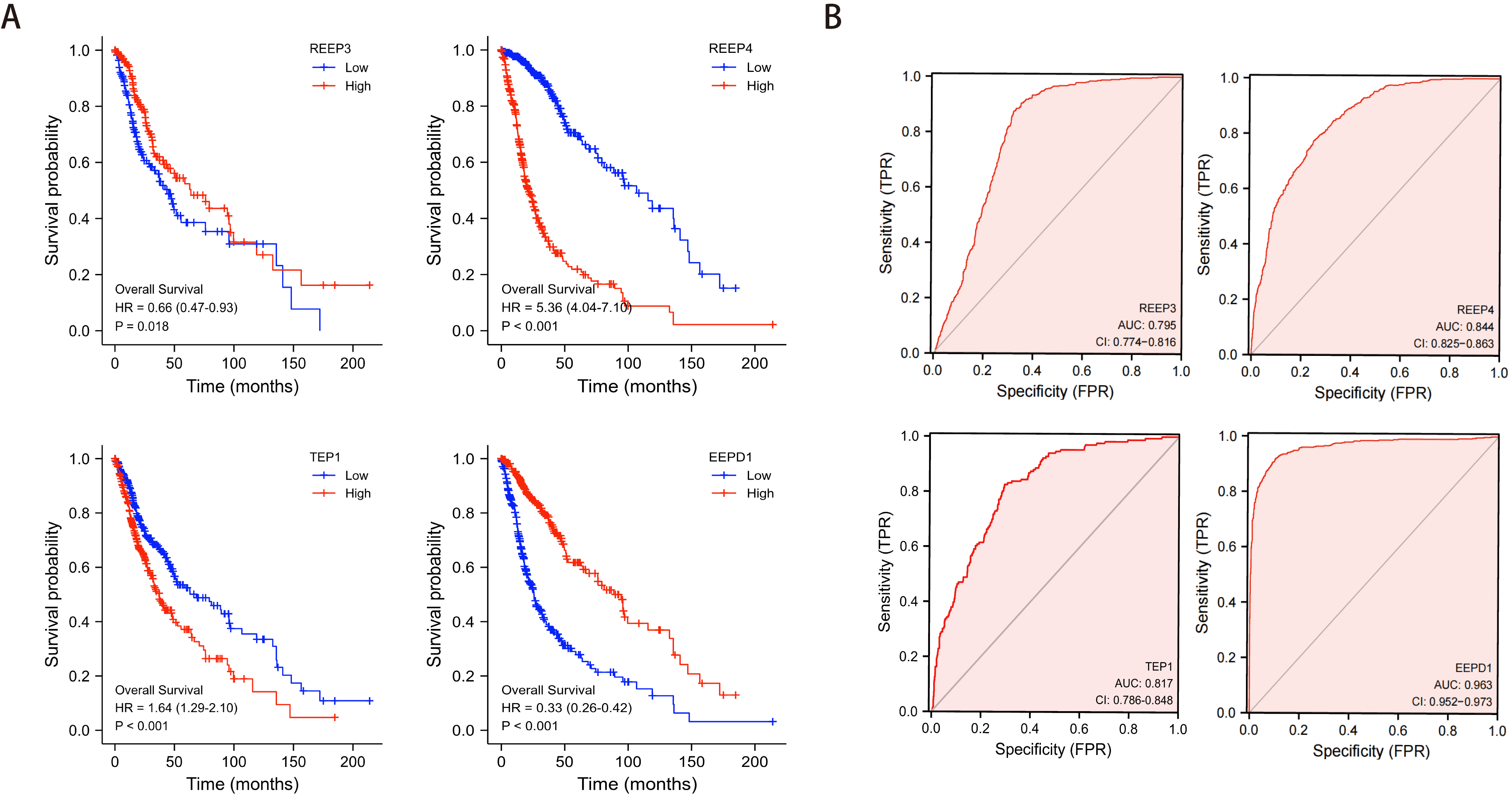


**Supplementary Figure 6.** The expression of EEP3, REEP4, TEP1, and EEPD1is associated with prognosis of GBM (A) Kaplan-Meier survival curves showing the overall survival (OS) for high and low expression levels of REEP3, REEP3, TEP1 and EEPD1 in GBM patients. The faster the curve drops, the worse the prognosis and vice versa. If hazard ratio (HR)>1, the high expression of the protein is associated with poor prognosis. If HR<1, the high expression of protein of interest is associated with good prognosis. If HR=1, the subject of interest doesn’t affect the survival time; (B) Analysis of the discriminative power of REEP3, REEP3, TEP1 and EEPD1 in GBM. The area under the curve for REEP3 is 0.795, REEP4 is 0.844, TEP1 is 0.817, EEPD1 is 0.963. The closer the AUC is to one, the more accurate of a diagnostic test. AUC of 0.5-0.7, 0.7-0.9, >0.9 indicates a low, medium, and high level of diagnostic power, respectively. FPR: false positive rate; TPR: true positive rate.
